# Novel biomanufacturing platform for large-scale and high-quality human T cells production

**DOI:** 10.1186/s13036-019-0167-2

**Published:** 2019-04-23

**Authors:** Jianfa Ou, Yingnan Si, Yawen Tang, Grace E. Salzer, Yun Lu, Seulhee Kim, Hongwei Qin, Lufang Zhou, Xiaoguang Liu

**Affiliations:** 1 Department of Biomedical Engineering, University of Alabama at Birmingham (UAB), 1670 University Blvd, Birmingham, AL 35233 USA; 2 Department of Cell, Developmental and Integrative Biology, University of Alabama at Birmingham (UAB), 1670 University Blvd, Birmingham, AL 35233 USA; 3 Department of Medicine, University of Alabama at Birmingham (UAB), Birmingham, AL 35294 USA

**Keywords:** Human T cells, Biomanufacturing platform, Stirred-tank bioreactor, Robust, High-quality and large-scale production

## Abstract

The adoptive transfer of human T cells or genetically-engineered T cells with cancer-targeting receptors has shown tremendous promise for eradicating tumors in clinical trials. The objective of this study was to develop a novel T cell biomanufacturing platform using stirred-tank bioreactor for large-scale and high-quality cellular production. First, various factors, such as bioreactor parameters, media, supplements, stimulation, seed age, and donors, were investigated. A serum-free fed-batch bioproduction process was developed to achieve 1000-fold expansion within 8 days after first stimulation and another 500-fold expansion with second stimulation. Second, this biomanufacturing process was successfully scaled up in bioreactor with dilution factor of 10, and the robustness and reproducibility of the process was confirmed by the inclusion of different donors’ T cells of various qualities. Finally, T cell quality was monitored using 12 surface markers and 3 intracellular cytokines as the critical quality assessment criteria in early, middle and late stages of cell production. In this study, a new biomanufacturing platform was created to produce reliable, reproducible, high-quality, and large-quantity (i.e. > 5 billion) human T cells in stirred-tank bioreactor. This platform is compatible with the production systems of monoclonal antibodies, vaccines, and other therapeutic cells, which provides not only the proof-of-concept but also the ready-to-use new approach of T cell expansion for clinical immune therapy.

## Introduction

Human T cell immunotherapies require high-quality and high-capacity cellular biomanufacturing. Current protocol for expanding tumor infiltrating T cells [1, 2] or CAR-T cells [3, 4] adopts the use of either traditional shaker flasks, gas-permeable GE WAVE bag [5, 6] or G-Rex bag [7]. The reported WAVE system generated 100–700 folds of T cell expansion from a 18-day perfusion culture [8–10], and the G-Rex system achieved up to 135-fold cell expansion from a 23-day batch culture [11].

Despite these technological advancements, the current T cell biomanufacturing process presents several weaknesses: 1) low efficiency of oxygen and nutrient transfer results in heterologous cellular metabolism, low cell viability and poor product quality; 2) ineffective process parameter control causes low bioprocess robustness; 3) lack of checkpoints in the early and middle stages of biomanufacturing for precise quality control limits the integrity and reproducibility of T cells for potential clinical use.

An advanced cellular biomanufacturing platform using stirred-tank bioreactor to produce high-quality and large-scale human T cells could overcome these technical challenges. Compared to WAVE bag, the stirred-tank bioreactor has the advantages of efficient mass transfer of oxygen and nutrients, high robustness of bioproduction, and outstanding scalability due to the precise process control of pH, temperature, dissolved oxygen (DO), agitation, gas sparging, and nutrients feeding. The stirred tank has been used to produce antibodies [12], biochemicals [13], viruses, hiPSCs, hiPSC-derived cardiomyocytes, and other biologics in our lab, which consistently shows very solid and robust bioproduction capability.

The objective of this study was to develop a novel fed-batch cellular biomanufacturing process, aiming to produce > 2000 × 10^6^ high-quality T cells with a short timeline. Various bioproduction process parameters, such as the bioreactor parameters of pH, DO, temperature, agitation and others, media, supplements, stimulation, seed age, and donors, were investigated to identify the key process regulators. The developed biomanufacturing process was also successfully scaled up in stirred-tank bioreactor. At multiple key stages of biomanufacturing, T cell quality was evaluated by monitoring the viable cell density, viability, T cell surface markers (total of 12) and T cell cytokines (total of 3). The accomplishments detailed in our study could significantly advance the bioproduction of human T cells and chimeric antigen receptor (CAR)-T cells and their application in immunotherapy.

## Results

### Efficient T cell biomanufacturing in stirred-tank bioreactor

**Fig. 1:**
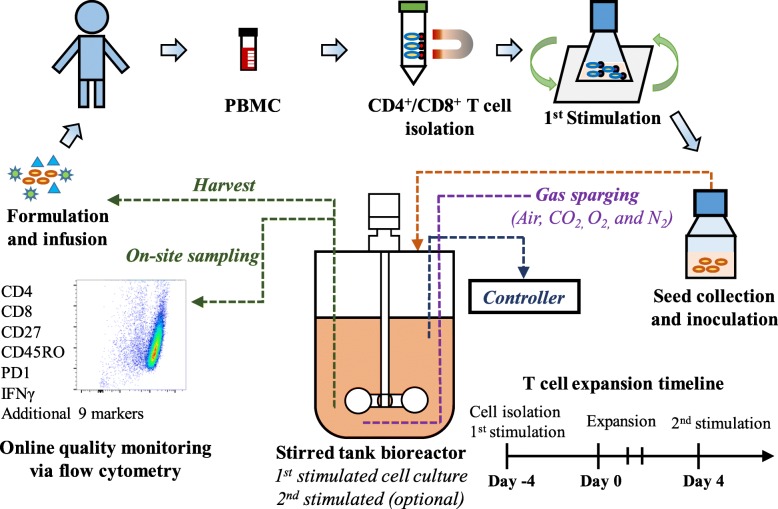
Diagram of scalable human T cell biomanufacturing in stirred-tank bioreactor. Seed culture was carried out in shaker flask for 1st stimulation and cell expansion until enough cells were generated to inoculate bioreactor. Each bioreactor culture was initiated on Day 0 and allowed cell expansion for 4 days. The 2nd stimulation (optional) was performed in bioreactor or shaker flask

The overview for our biomanufacturing process is detailed in Fig. 1. Briefly, human peripheral blood mononuclear cells (PBMCs) were used to isolate CD4^+^ or CD8^+^ T cells with magnetic beads. The purified T cells were stimulated with anti-CD3 and anti-CD28 mAbs for four days to prepare the seed culture for processing in the stirred-tank bioreactor. Process parameters, such as temperature, pH, agitation, DO, gas sparging, and cytokine supplementation, were precisely controlled. An optional second stimulation in the bioreactor extended T cell culture longevity and increased production capacity. The bioreactor cultures were sampled daily to monitor cell growth and quality in the early, middle and late stages of T cell production. In this study, the stirred-tank bioreactor-based human T cell biomanufacturing process was developed by evaluating multiple cell culture factors, such as basal media, medium supplements, seed culture, stimulation strategy, scale-up, and donor variability. Our optimized cell expansion timeline is also shown in Fig. 1, where human T cells were purified and stimulated on Day − 4, expanded into growth medium with supplement on Day 0, and harvested or re-stimulated on Day 4.

### Basal media and supplements

**Fig. 2:**
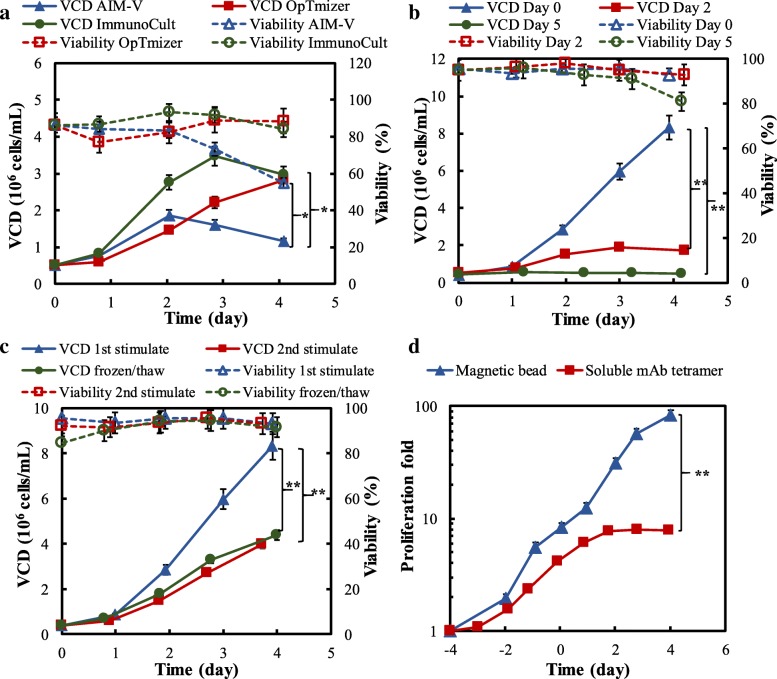
Human T cell biomanufacturing development. **a** Effect of basal media on T cell growth. **b** Cell proliferation with the seeding culture collected from Days 0, 2 and 5. **c** Proliferation capability of cells post 1st stimulation, 2nd stimulation and freeze/thaw. **d** Re-stimulation with anti-CD3/CD28 mAbs coated on solid beads or mAbs tetramer. OpTmizer basal medium was used and 30 IU/mL IL-2 was fed on Days 0, 2, and 4. *p < 0.05, *p < 0.01. *P*-values were analyzed by one-way ANOVA. The error bars are presented as standard error of the mean (SEM)

The maintenance and expansion of T cells is highly dependent on culture media and other factors. To reduce the risk of disease transmission and cellular product variability caused by animal derived components, three serum-free, xeno-free and animal origin-free media, i.e., AIM-V, OpTmizer and ImmunoCult, were evaluated. The seed culture was scaled up in AIM-V medium for 3 days in T75 flask after removing the stimulation magnetic beads. As shown in Fig. 2a, the maximal viable cell density (VCD) in ImmunoCult and OpTmizer media was similar (2.83 × 10^6^ and 3.49 × 10^6^ cells/mL), but higher than that in AIM-V medium (1.90 × 10^6^ cells/mL). In addition to cell growth, OpTmizer medium maintained consistent phenotype and function of T cells produced from the high-density cell culture (data described in following sections) and the cost of raw material was lower than the other two media, so OpTmizer was used as the basal medium throughout this study. The cytokine interleukin-2 (IL-2) is critical to a healthy T cell growth due to its important regulatory role in cell survival, proliferation, and differentiation [14, 15]. The level of endogenous IL-2 is not high enough to support the high-density and rapid cell expansion rate [16], therefore basal media was supplemented with 30 IU/mL IL-2 in this study. Amino acid supplement was also evaluated in this study but had no effect on T cell growth (data not shown).

### Seed culture and stimulation

The evaluation of seed age post 1st stimulation showed that the VCD_max_ of T cell cultures using the seed cells collected on Days 0, 2 and 5 were 8.33, 1.89, and 0.55 × 10^6^ cells/mL, respectively (Fig. 2b). Similar to in vivo T cell growth [17, 18], the youngest seed cells had the lowest doubling time (Td) of 13.0 ± 0.2 h (Table 1). It is noted that the cells collected on Day 5 post 1st stimulation almost lost proliferative capacity, with Td of 99.5 h. To extend culture longevity and T cell expansion capability, the effects of re-stimulation (2nd stimulation on Day 4) on cell expansion and function were evaluated. In practical application, the isolated or harvested T cells might need freeze for storage or transportation, so we also evaluated the effect of T cell freeze/thaw on cell growth. As shown in Fig. 2c, T cell proliferation post 2nd stimulation was only 50% of post 1st stimulation, with VCD_max_ of 3.98 vs. 8.33 × 10^6^ cells/mL. However, the freeze/thaw of T cells collected on Day 0 did not reduce cell proliferation significantly after re-stimulation, with VCD_max_ of 4.38 × 10^6^ cells/mL (data not shown). Despite the decreased proliferation ability post re-stimulation, e.g. 500-fold after 2nd stimulation vs 1000-fold after 1st stimulation, serial stimulation resulted in as much as up to 500,000-fold cell expansion (Table 1).

**Table 1:** Summary of key parameters in human T cell biomanufacturing

Biomanufacturing Parameters^a,b,c^		Scale (mL)	VCD_max_^d^ (10^6^ cells/mL)	TVC (10^6^ cells)	Viability^d^ (%)	μ^d^(hr^− 1^)	Td^c^(hr)
Medium	AIM-V	30	1.90 ± 0.11	56.9 ± 3.2	76.8 ± 2.5^e^	0.030 ± 0.001	23.2 ± 0.8
	OpTmizer	30	2.83 ± 0.12	84.8 ± 3.6	87.5 ± 1.4^e^	0.030 ± 0.004	23.1 ± 2.9
	ImmunoCult	30	3.49 ± 0.52	104.6 ± 15.5	91.8 ± 1.1	0.041 ± 0.000	17.1 ± 0.2
Seed and stimulation	1st stimulated Day 0 seed	80	8.33 ± 0.11	666.0 ± 8.5	93.0 ± 1.4	0.053 ± 0.001	13.0 ± 0.2
	1st stimulated Day 2 seed	80	1.89 ± 0.04	151.2 ± 3.4	95.0 ± 1.4	0.029 ± 0.002	23.8 ± 1.5
	1st stimulated Day 5 seed	80	0.55 ± 0.02	44.1 ± 2.0	96.0 ± 1.4	0.007 ± 0.001	99.5 ± 17.2
	2nd stimulated Day 0 seed	80	3.98 ± 0.06	318.4 ± 4.5	93.0 ± 0.0	0.042 ± 0.002	16.7 ± 0.7
Scale-up^f^	T75	10	4.05 ± 0.13	40.5 ± 1.3	90.5 ± 0.7	0.034 ± 0.002	20.4 ± 0.9
	SF125	30	3.65 ± 0.18	109.4 ± 5.3	91.5 ± 2.1	0.041 ± 0.001	16.9 ± 0.4
	Spinner	80	3.98 ± 0.06	318.4 ± 4.5	93.0 ± 0.0	0.042 ± 0.002	16.7 ± 0.7
	Bioreactor	800	6.40 ± 0.46	5120.0 ± 367.7	94.0 ± 1.4	0.046 ± 0.002	15.2 ± 0.7

Notes

^a^The human T cell biomanufacturing was performed in spinner flasks for 4 days with basal medium of OpTmizer, feed of 30 U/mL IL-2 and 1st stimulation on Day 0 and Day 2, unless otherwise specified.

**Fig. 3:**
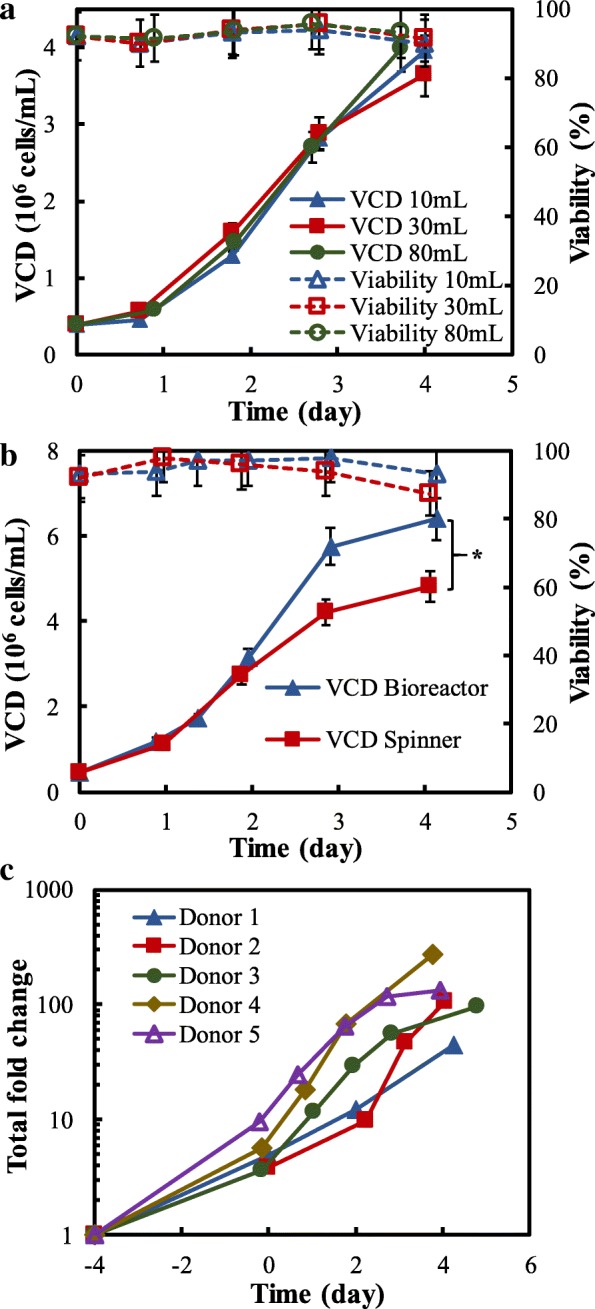
Robustness and capability of the developed human T cell biomanufacturing. **a** Comparison of culture volume and container using the re-stimulated CD4^+^ T cell. **b** Large-scale culture in 2-L stirred-tank bioreactor using the 1st stimulated CD4^+^ T cell. **c** Total cell expansion fold was consistent among various donors. OpTmizer medium was used in the evaluation supplemented with 30 IU/mL IL-2 on Days 0, 2, and 4. *p < 0.05, **p < 0.01. *P*-values were analyzed by one-way ANOVA. The error bars are presented as SEM

^b^Samples from multiple donors were used in the biomanufacturing process development, and T cells isolated from the same donor were used in the evaluation of one biomanufacturing parameter (i.e., medium, seed and stimulation, and scale-up). Significant difference between bioreactor runs in triplicate with *p* ≤ 0.05 was considered in the two-tailed t test. The results of cross donor comparison are presented in Fig. 3.

^c^All data are presented as mean ± standard error of the mean (SEM).

^d^The variation in cell growth parameters, such as VCD, viability and cell growth rate, was caused by the different biomanufacturing process parameters.

^e^The viabilities of the T cells when reaching maximal VCD are presented. The relatively low harvest viability in the medium evaluation study was caused by an extended seed culture process in the early stage of process development, i.e. 6 days instead of 4 days.

^f^The scale-up strategy was designed following the procedure described in Fig. 1.

In addition to multiple stimulations, we also tested two forms of stimulating anti-CD3 antibody and anti-CD28 antibody, including magnetic bead and soluble tetramer. Both stimulators benefited T cell proliferation but the mixture of two individual mAbs on beads had higher efficiency than their tetramer in liquid (Fig. 2d). Taken from the experimental results, the ideal stimulators should achieve homogenous culture with good mass transfer rate, serial stimulation in bioreactor, and robust and scalable cellular biomanufacturing. Additionally, we can improve the expansion of antigen-specific T cells by optimizing the ratio between anti-CD3 mAb and anti-CD28 mAb.

### Biomanufacturing scale-up and robustness

The scale-up capability of our human T cell biomanufacturing platform was evaluated in both small and large-scale culture. Firstly, the T cell proliferation from cultures in 10-mL T-flask, 30-mL shaker flask and 80-mL spinner flask was compared. We found that these cultures had similar VCD_max_ of 3.65–4.05 × 10^6^ cells/mL on Day 4 (Fig. 3a) but different viability, i.e., 52, 80 and 83% on Day 6 (data not shown). The higher viability in shaker and spinner flasks could be attributed to the higher oxygen transfer rate. Secondly, we expanded T cells in a 2-L stirred-tank bioreactor with working volume of 800 mL (Fig. 3b). With the automatic process control of pH, DO and gas sparging, we produced T cells with VCD_max_ of 6.40 × 10^6^ cells/mL and a high purity (av. > 95%) of CD3^+^CD4^+^ or CD3^+^CD8^+^ T cells (Table 2). These data demonstrate that the established stirred-tank bioreactor-based biomanufacturing platform can be scaled up to produce a large amount of T cells. Additionally, we tested the robustness and reproducibility of this process by cultivating T cells from 5 donors (Fig. 3c), and found that the total proliferation fold was in the range of 132–1011 with single stimulation. It is obvious that our new human T cell biomanufacturing has multiple advantages compared to traditional biomanufacturing process, including high VCD (> 6.0 × 10^6^ cells/mL), proliferation ability (> 100 folds) and short production timeline (4 days).

**Table 2:** Online quality assessment of human CD4^+^ and CD8^+^ T cells

Function^a,b,c^	Markers	Day − 4^d^		Day 0		Day 2		Day 4		Day 8 (re-stimulated)	
		CD4^+^	CD8^+^	CD4^+^	CD8^+^	CD4^+^	CD8^+^	CD4^+^	CD8^+^	CD4^+^	CD8^+^
Cell type	CD4^+^	99.1 ± 0.5	0.7 ± 0.0	99.0 ± 1.1	0.8 ± 0.0	99.6 ± 0.2	0.6 ± 0.3	99.6 ± 0.4	0.4 ± 0.2	98.6 ± 1.1	0.3 ± 0.0
	CD8^+^	0.8 ± 0.1	99.5 ± 0.0	0.4 ± 0.3	99.8 ± 0.0	0.3 ± 0.1	99.3 ± 0.0	0.4 ± 0.4	93.8 ± 0.0	0.4 ± 0.1	99.0 ± 0.1
Activation signal	CD3^+^ (stimulation)	97.8 ± 2.6	98.2 ± 2.2	97.5 ± 2.8	92.6 ± 8.4	99.4 ± 0.7	96.3 ± 4.1	98.3 ± 2.3	91.7 ± 7.7	95.1 ± 3.7	96.8 ± 0.5
	ICOS^+^/CD278^+^ (co-stimulation)	30.3 ± 19.9	13.3 ± 10.8	99.6 ± 0.1	99.5 ± 0.6	99.7 ± 0.2	99.2 ± 1.0	99.5 ± 0.6	91.2 ± 12.7	99.8 ± 0.2	99.8 ± 0.2
	OX40^+^/CD134^+^ (co-stimulation^e^)	1.8 ± 1.3	0.1 ± 0.1	2.7 ± 1.6	1.7 ± 1.3	0.2 ± 0.2	0.1 ± 0.1	0.3 ± 0.5	0.1 ± 0.1	0.4 ± 0.3	0.0 ± 0.0
	CD27^+^ (co-stimulation)	83.3 ± 2.7	64.1 ± 5.1	83.1 ± 9.9	95.4 ± 1.1	87.9 ± 4.7	86.6 ± 4.0	92.3 ± 3.4	89.7 ± 1.3	67.2 ± 2.4	88.5 ± 1.7
Inhibitory signal	PD-1^+^/CD279^+^ (TCR signaling)	5.7 ± 4.3	6.5 ± 5.6	84.5 ± 4.3	84.7 ± 4.3	30.3 ± 24.6	7.0 ± 3.8	8.1 ± 3.9	3.3 ± 2.5	63.1 ± 6.0	67.9 ± 15.6
	LAG-3^+^/CD223^+^	95.0 ± 6.2	97.6 ± 2.8	23.3 ± 11.9	61.6 ± 15.8	11.0 ± 5.8	24.0 ± 12.7	4.4 ± 1.2	4.9 ± 4.0	16.4 ± 8.6	47.4 ± 8.6
	KLRG1^+^	0.9 ± 0.4	1.4 ± 0.7	5.5 ± 2.5	7.9 ± 2.3	7.5 ± 0.7	6.6 ± 1.0	6.7 ± 2.3	6.4 ± 3.4	6.4 ± 1.5	4.7 ± 0.4
Memory type^f^	CCR7^+^ (central memory T cell)	19.4 ± 7.6	6.0 ± 1.0	90.7 ± 6.6	86.5 ± 8.0	78.3 ± 5.8	70.1 ± 5.2	81.7 ± 5.5	68.5 ± 6.4	39.1 ± 9.9	29.2 ± 7.2
	CD45RO^+^ (memory T cell)	86.7 ± 4.0	57.4 ± 13.6	97.0 ± 4.4	99.7 ± 0.4	98.4 ± 1.9	93.5 ± 7.8	98.6 ± 0.9	94.1 ± 2.6	99.6 ± 0.3	99.0 ± 0.1
	CD45RA^+^ (naïve T cell)	13.9 ± 3.0	55.3 ± 6.6	2.4 ± 1.1	3.5 ± 2.6	3.7 ± 2.3	3.6 ± 2.4	0.7 ± 0.3	1.1 ± 0.7	1.9 ± 1.5	3.4 ± 0.9

Notes

^a^The CD4^+^ T cells and CD8^+^ T cells were cultivated in single culture. The T cells used for data collection came from 3 donors.

^b^Flow cytometry was used to access T cell quality and the expression of these markers was reported as the binding intensity of antibodies.

^c^All data are presented as mean ± standard error of the mean (SEM).

^d^Day-4 data was collected from isolated cells without stimulation. Day 8 data was collected after 2nd stimulation.

^e^Co-stimulation could happen with various mechanisms^25^. The low values of OX40/CD134 indicated the low possibility of this receptor correlated co-stimulation mechanism.

^f^All cells obtained a memory phenotype after stimulation.

### Co-culture of CD4^+^ and CD8^+^ T cells

**Fig. 4:**
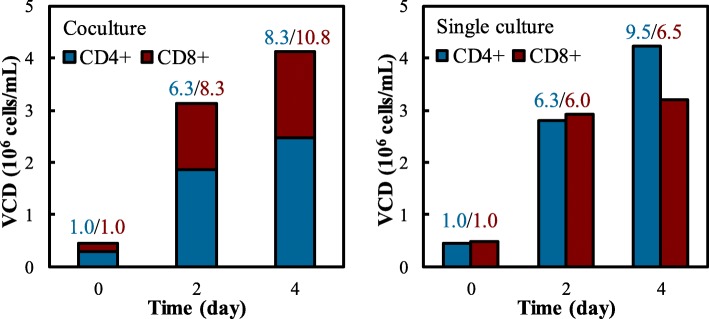
Biomanufacturing of the co-culture and single culture of CD4^+^ and CD8^+^ cells. The CD8^+^ cells grew faster in the co-culture of CD4^+^ and CD8^+^ T cells than the single culture. The values of cell expansion fold were presented, which were calculated using the VCD of Day 0 culture as baseline. Culture conditions: 4-day seed culture with one stimulation was used; Day 0 was defined as the inoculation time of bioreactor runs; OpTmizer medium and bioreactor parameters of 37 °C, pH 7.4, DO 70%, agitation 70 rpm and gas sparging rate 0.01 VVM were used. *p < 0.05. The *p*-values were analyzed by one-way ANOVA. The error bars are presented as SEM

To understand how the interaction between CD4^+^ and CD8^+^ T cells affected cell proliferation in our stirred-tank bioreactor-based biomanufacturing process, we compared the cell growth of single CD4^+^ T, single CD8^+^ T, and co-cultivated CD4^+^ T and CD8^+^ T cells with an initial ratio of 2:1 (Fig. 4). It is found that CD8^+^ T cells grew faster than CD4^+^ T cells, with the reduced CD4^+^/CD8^+^ ratio from 2:1 on Day 0, 1.5:1 on Day 2 and 1.5:1 on Day 4, and total VCD_max_ of 4.38 × 10^6^ cells/mL on Day 4. Differently from co-culture, the single culture of CD4^+^ cells or CD8^+^ cells had similar cell growth rate from Day 0 to Day 2, and CD4^+^ cells continued growing while CD8^+^ stopped growing from Day 2 to Day 4. According to literature, CD4^+^ T cells divide rapidly and secrete cytokines to assist the initial priming and activation of the immune response of CD8^+^ T cells to antigen [19, 20] and improve their cytotoxicity [21, 22] when T helper cells are activated. In this study, we found that CD8^+^ T cells proliferated in the co-culture with CD4^+^ T helper cells had faster cell growth than single culture, which can be explained by the interaction between these two cell types. Different from CD8^+^ T cells, the cell growth rate of CD4^+^ T cells was similar in co-culture and single culture.

### Human T cell quality assessment

To transit from bench to bedside for cellular therapy, process validation is required to establish scientific evidence that this process is capable of consistently delivering high-quality products. It is commonly required to assure product identity, purity, safety and potency using various tests, including cell surface markers (identification), cytokine production capacity (function and potency), sterility, and absence of endotoxin, mycoplasma and viruses [23, 24]. In this study, we evaluated the surface and intracellular markers correlated with T cell activation, inhibition, memory, and cytokine signaling. All the surface protein analysis data indicated that the produced T cells were functional after expansion.

#### T cell activation signal

**Fig. 5:**
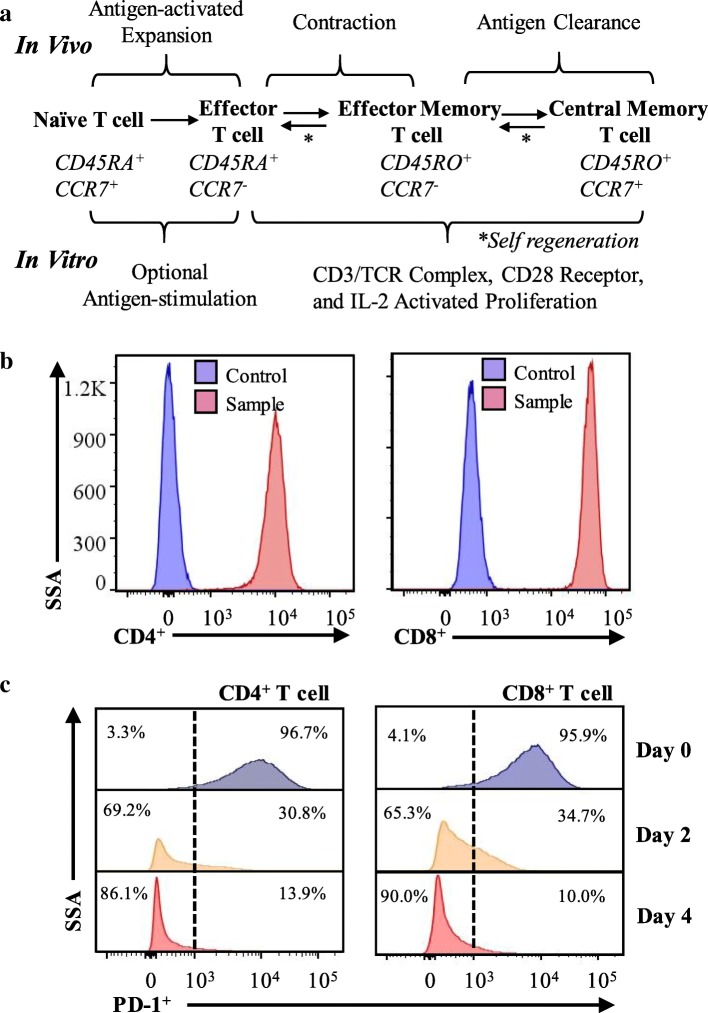
Quality of the T cells in our novel biomanufacturing. **a** Diagram of T cell proliferation and differentiation pathway. **b** Both CD4^+^ and CD8^+^ T cells showed high 99% purity after the expansion. **c** Proliferation signal. PD-1 expression level was related to cell proliferation status. **Note:** The CCR7, CD45RA and CD45RO surface proteins were used to identify cell subsets [57]

As summarized in Fig. 5, effector T cells, effector memory T cells, and central memory T cells proliferated when instructed by the CD3/T cell receptor (TCR) and CD28 receptor. This study evaluated multiple T cell activation signaling receptors, including CD3, ICOS/CD278, OX40/CD134 and CD27. It is reported that T cells response to anti-CD3 resembles antigen activation [25], where the enzymatically cleaved antigen peptides bind to the TCR. The CD28 co-stimulation provides an essential expansion signal by promoting the survival of lymphocytes [26]. In this study, we found that the expression of CD3 was high (91.7–99.4%) in both CD4^+^ and CD8^+^ T cells during the entirety of the biomanufacturing (Table 2). Additionally, CD4^+^ and CD8^+^ T cells have high purity (> 99%) after expansion (Fig. 5b). These data supported the biological evidence for the high proliferation rate of T cells and further indicated that our T cell biomanufacturing platform enables powerful cell proliferation. The expression level of the co-stimulation receptor, inducible T cell co-stimulatory signaling receptor (ICOS/CD278), was significantly elevated and maintained at > 99% post CD3/CD28 stimulation from 13.3–30.3% pre-stimulation, which can improve the efficiency of immunotherapy [14]. As expected and consist with previous reports [25, 27, 28], the expression of CD27 (64.1–95.4%) had little or no change during T cell expansion. The T cells have multiple co-stimulation and co-inhibition molecular mechanisms [25]. The low expression of OX40/CD134 (0.0–2.7%), type V family receptor, indicated the low possibility of OX40/CD134 correlated co-stimulation mechanism in the T cells expanded in our biomanufacturing.

#### T cell inhibition signal

We tested the expression level of three inhibitory signaling receptors, i.e., PD-1/CD279, LAG-3/CD223, and KLRG1 at multiple stages of the stirred-tank bioreactor cellular biomanufacturing. PD-1/CD279 is an inhibitory receptor that can prevent the T cells from proliferating. The upregulated expression of programmed cell death protein 1 (PD-1/CD279) on Day 0 (start point of the T cells expansion) and Day 8 (harvest time point) indicated poor proliferation ability of the cultivated T cells (Fig. 5c), which may be upon TCR antigen activation or CD3 bypass [29–31] and correlate with the strength of TCR signaling [32]. The PD-1 expression increased from 5.7–6.5% on Day − 4 to 84.5–84.7% on Day 0, and increased from 3.3–8.1% on Day 4 to 63.1–67.9% after 2nd stimulation on Day 8. These data indicated that CD3/CD28 stimulation significantly up-regulated the expression of PD-1. Additionally, previous studies showed that PD-1 is also a suppressive molecule in cell cycle control [33], and its expression is low or moderate in healthy human T cells [34]. It was noted in this study that the PD-1 expression rapidly decreased to 7.0–30.3% on Day 2 and 3.3–8.1% on Day 4, indicating the suppression of PD-1. The expression of lymphocyte activation gene 3 protein (LAG-3), an inhibitory receptor involved in T cell exhaustion [35] and releasing suppressive signal [25], decreased from 95.0–97.6% on Day − 4 to 23.3–61.6% on Day 0 and 4.4–4.9% on Day 4. The expression of killer cell lectin-like receptor subfamily G member 1 (KLRG1), a reported inhibitory signal receptor [36], was low (0.9–7.9%) during cell expansion. The overall percentage of the exhausted T cells (PD-1^+^LAG-3^+^KLRG1^+^) was < 5% before and after cell expansion in the biomanufacturing.

#### Memory T cell/naïve T cell

Monitoring the memory cell type is very important to quality control because it could be changed by stimulation and cell expansion (Table 2). The donors’ ages and health condition can also change the ratio of memory T cells/naïve T cells [37], and the central memory T cell can differentiate to effector memory T cells [38, 39]. This study tested the expression of three memory T cell receptors including CCR7, CD45RO and CD45RA in T cells derived from same donor. As presented in Table 2, the kinetic expression profiles of these three markers were different endogenously throughout the biomanufacturing process. Specifically, CCR7 reached peak expression on Day 0 then slightly reduced from Day 0 to Day 4, suggesting that the population of central memory cells [40] was reduced during T cell culture. The marker CD45RO maintained high expression level during expansion, while the expression of CD45RA significantly decreased from Day − 4 to Day 0 then was attenuated until Day 8. Elevated CD45RO expression indicated the fast cell growth of effector memory T cells [41, 42] in our stirred-tank bioreactor. The CD45RA^+^_Low_CD45RO^+^_High_ population suggested that our cultivated T cells adopted a memory phenotype after activation. The low cell growth of naïve cell could be caused by the lack of additional antigen stimulation and cytokines (e.g., IL-4, IL-7, and IL-15) [43].

#### Evaluation of cellular function

We evaluated the function of T cells by measuring the levels of their intracellular cytokines (i.e., IFN-γ, IL-2, and IL-4) at 1st stimulation, inoculation, and harvest. The CD4^+^ T-helper cells were divided into T-helper type 1 (Th1) and type 2 (Th2) cells. IFN-γ is the signature cytokine generated by Th1 to activate macrophage; IL-4 is a signature cytokine of Th2 associated with strong antibody response [44, 45]; both Th1 and Th2 can produce IL-2 [46]. The change of signature cytokine expression indicated the population of Th1 type CD4^+^ T helper cells decreased from Day − 4 to Day 0. As shown in Table 3, the production of IFN-γ by CD4^+^ T cells decreased by ~ 50% from Day − 4 to Day 0 then maintained low level production during expansion in bioreactor; the IL-2 level increased from Day − 4 to Day 0 then maintained elevated values; and IL-4 production level was constantly low during the biomanufacturing. These data indicated that Th1 cell population was reduced post stimulation, and Th2 population had no obvious change during the cellular biomanufacturing, indicating the possible change of T cell function against cancer [47, 48]. The IFN-γ can promote CD8^+^ T cell motility and enhance the cytotoxicity against tumor and virus [49], and the memory CD8^+^ T cell is a major source of IFN-γ [50]. Therefore, the high IFN-γ expression in CD8^+^ T cells indicated that CD8^+^ remained cytotoxic after expansion. The high IL-2 expression in CD8^+^ T cells can increase the antigen response [51] and induce the secretion of proinflammatory cytokines (e.g., IL-6, IL-1b, IFN-γ, and TNF-α) [16] for cytotoxic function.

**Table 3:** Cytokine production by T cells expanded in stirred-tank bioreactor

Data source	Cytokines secretion^a^	Day -4		Day 0		Day 4	
		CD4^+^	CD8^+^	CD4^+^	CD8^+^	CD4^+^	CD8^+^
This study^b^	IFN-γ	46.2–54.2	50.9–62.0	15.6–20.8	20.3–34.8	21.1–31.1	39.8–50.9
	IL-2	64.9–76.7	19.8–39.5	79.8–95.6	77.2–93.8	75.7–95.9	74.5–96.1
	IL-4	4.6–6.5	1.7–3.5	4.2–8.7	10.2–19.9	6.0–9.1	5.2–8.2
Literature	IFN-γ	17–23 [64] 6.7–38.9 [65]	27–37 [64] 20–58.4 [65]	N/A		23-47^b^ [66] 15-41^d^ [67]	42-72^b^ [66] 4–42 [68] 10-41^d^ [67]
	IL-2	60 [64]	25–35 [64]			53-78^d^ [67]	49-97^d^ [67]
	IL-4	0.2–4.9 [65]	0.1–1.6 [65]			19-47^c^ [66]	0-2^b^ [66]

Notes

^a^All data are presented as mean ± standard error of the mean (SEM).

^b^Data were collected using the T cells isolated from 3 donors.

^c^Data were collected from a 21-day culture.

^d^Data were collected from a 14-day culture.

## Discussion

In this study, we successfully developed a robust, scalable, stirred-tank bioreactor-based cellular biomanufacturing platform to generate large-quantity and high-quality human CD4^+^ and CD8^+^ T cells.

### New human T cell biomanufacturing

Compared to the GE WAVE system currently used in large-scale cell culture of T cells or other therapeutic cells, our T cell biomanufacturing has several advantages. First, the perfusion culture in WAVE takes 18 days to expand by > 100 folds from stimulation to harvest [15], [52, 53], but our stirred-tank bioreactor can achieve 100-fold expansion within 8 days. Second, the agitation and gas sparging in the stirred-tank bioreactor offered optimal oxygen transfer, nutrients transfer, and a more homogenous environment without Dynabeads. Third, our system can use a glass vessel or disposal plastic bag with the same controller that precisely controls various process parameters, which is compatible with the production systems of other therapies such as monoclonal antibodies, vaccines, and stem cells-derived therapeutic cells. This biomanufacturing platform provides not only the proof-of-concept but also the ready-to-use new approach of T cell expansion for clinical immune therapy. Fourth, the utilization of serum-free cell culture in our process reduces the risk of disease transmission and cellular product variability caused by animal derived component.

### Scalable and robust biomanufacturing

Our T cell biomanufacturing yielded very consistent data regarding to cell growth rate, doubling time and cell quality at both small and large scales. Moreover, the inclusion of different donors’ T cells in our study confirmed that our bioreactor platform can be used to expand T cells of various qualities. In addition, our study showed that the second stimulation further improved T cell expansion although the proliferation ability was reduced. However, the repeated stimulation may potentially reduce T cell function against infections and tumors [35], and thereby decrease their therapeutic quality. It is worthy of further investigating the effect of multiple stimulation in future studies.

### Stimulation strategy

Various stimulation strategies have been previously reported for obtaining different T-cell effector subsets. For example, IFN-γ plus IL-12 led to Th1 cell differentiation, IL-4 helped Th2 cell development, while IL-4 plus TGF-β resulted in Th9 cells [54]. It was reported that the central memory T cells showed higher anti-tumor toxicity than the effector memory T cells in vivo [55, 56], and the stimulation by combined anti-CD3, anti-CD28, IL-7 and IL-15 was reported to improve T cell specific cytotoxicity [57]. Refining the T cell to a specific phenotype, a.k.a. T cell polarization, is a cutting edge of clinical research [58], and further evaluation is needed in our new biomanufacturing platform.

### Online quality assessment

In addition to the large-quantity rapid human T cell expansion (i.e. > 5 billion T cells within 4 days), our biomanufacturing platform used cell growth, viability, surface markers, and cytotoxic function as the critical quality assessment criteria. First, we evaluated T cell quality using 12 surface markers and 3 intracellular cytokines in early, middle and late stages of our biomanufacturing. Since the maintenance of cell product comparability and quality ultimately relies on the production process consistency, the multiple checkpoints for critical quality attributes could assure a high-quality therapeutic human T cells. It is imperative to identify potential product quality and process scale-up pain points in the early stage of T cell bioproduction, which has not been well established before our study. Second, it is important to harvest or re-stimulate human T cells before apoptosis happens. Previous studies determined the need for re-stimulation by cell size [15]. Our study indicated that we can use multiple activation signals, inhibitory signals, and cell type markers to predict the re-stimulation time point.

### Application and consideration in CAR-T biomanufacturing

In current clinical practice, human primary T cells such as tumor infiltrating T cells or CAR-T cells are expanded in traditional shaker flasks or gas-permeable bags. This study indicated that our novel stirred-tank bioreactor T cell biomanufacturing process can be applied to CAR-T cells production because our release criteria of cell viability and CD3^+^ marker are higher than the current CAR-T production in WAVE system, i.e. viability of > 90% vs. ≥70% and CD3^+^ cells of > 90% vs. ≥80%. Clinical trial or application reported severe in vivo cytokine storm post T cell infusion [59–61], including IL-2, IL-7, IL-15, IL-12, TNF-α, and IFN-γ [59]. The correlation between in vitro and in vivo cytokine assessment should be investigated in order to further improve the safety of CAR-T cellular therapy.

#### Future directions

A stimulation without targeting specific antigen was used in this study for proof of concept. In the next step, we will use this novel platform to engineer and produce CAR-T cells that target specific cancers. Moreover, we used the defined subpopulations of T cells, e.g. CD4^+^ and CD8^+^ T cells, to explore more details of cell quality in this study. In the future, we will compare the discovery to a leukapheresis mixture, which is currently used in CAR-T cell therapy, and guide the optimization of mixture culture cell quality. Moreover, we also plan to develop a closed cell harvest system to take the manufacturing one step closer to clinical practice [62]. Additionally, some cutting-edge technologies, such as replacing autologous T cells with new cell sources, will also benefit T cell manufacturing [63].

## Conclusions

In this study, we developed a new biomanufacturing platform to produce a reliable and reproducible large quantity of human T cells for immune cancer therapy. The accomplishment of our study will provide not only the proof-of-concept principle but also the ready-to-use bioproduction platform for a new means of T cell expansion for clinical immune cancer therapy.

## Methods

### Materials

All basal media, supplements and reagents used in this study were purchased from Thermo Fisher Scientific (Waltham, MA) unless otherwise specified.

### T cell isolation

Peripheral blood mononuclear cells (PBMCs) were provided by StemExpress (Folsom, CA) collecting from healthy donors under written informed consent. The PBMCs were obtained using Institutional Review Board (IRB) approved consent forms and protocols. CD4^+^ and CD8^+^ T cells were isolated using Dynabeads® CD4 and CD8 positive isolation kit following the manufacturing instructions, respectively. Generally, the PBMC were washed with buffer 1 (Ca^2+^ and Mg^2+^ free PBS, 0.1% BSA, 2 mM EDTA, pH 7.4) first, then incubated with magnetic beads on ice for 20 mins with gentle mixing to capture CD4^+^ and CD8^+^ T cells. Held on a magnet, the supernatant was removed, and the complex of beads-cells was washed with buffer 1 twice. The CD4^+^ or CD8^+^ T cells were dissociated from beads using detach reagent for 45 mins at room temperature, followed by removing magnetic beads with the magnet. The released T cells were washed with culture medium twice before cultivation.

### T cell stimulation and cell culture

The isolated CD4^+^ and CD8^+^ T cells were seeded at 0.5 × 10^6^/mL in T flask or shaker flask for stimulation. Two stimulations reagents were evaluated in this study: 1) Dynabeads® Human T-Activator CD3/CD28 beads were added to the culture at cell:bead ratio of 1:1 and incubated for 4 days. 2) ImmunoCult™ Human CD3/CD28 T Cell Activator (Stemcell Technologies, Vancouver, Canada) was added at 25 μL/mL culture. Three basal media were evaluated, including AIM-V Medium CTS, OpTmizer CTS and ImmunoCult™-XF T (Stemcell Technologies). The T cell cultures were supplemented with 30 IU/mL IL-2 on Day 0 unless otherwise specified. The stirred-tank bioreactor was controlled at 37 °C, pH 7.4, DO 70%, agitation 70 rpm and gas sparging rate 0.01 VVM. The shaker flask culture was maintained at 37 °C, 5% CO_2_ and 125 rpm, and the spinner flask culture was incubated at 37 °C, 5% CO_2_ and 50 rpm. The pH of all the cultures in flasks were adjusted to 7.4 once a day with 0.5 M Na_2_CO_3_. The cell expansion cultures were sampled daily to monitor cell growth by measuring the VCD and viability using Countess II automated cell counter and trypan blue (Thermo Fisher Scientific, Waltham, MA).

### Flow cytometry analysis

The following antibodies used in flow cytometry analysis were purchased from BioLegend (San Diego, CA): anti-CD8a (FITC), anti-CD45RO (PerCP-Cy5.5), anti-PD-1 (PE), anti-CCR7 (PE-Cy7), anti-CD27 (APC), anti-CD4 (APC-Cy7), anti-CD45RA (BV510), anti-CD223 (PerCP-Cy5.5), anti-CD3 (PE), anti-KLRG1 (PE-Cy7), anti-CD278 (APC), anti-CD4 (APC-Cy7), anti-CD134 (BV510), anti-IFN-γ (FITC), anti-IL-2 (PE), and anti-IL-4 (APC). The staining dyes were determined on the base of their fluorescent compatibility. Negative samples were prepared by subtracting one antibody from the panel at a time. In cell surface staining, cells were harvested and washed with staining buffer (PBS, 1% BSA) at 400 g for 7 mins, and treated with Fc Receptor Blocking Solution (Biolegend 1 μL/1 × 10^6^ cells) at 4 °C for 15 mins. Cells were then stained with antibodies and LIVE/DEAD Blue Dead Cell Stain at 4 °C for 30 mins, washed with staining buffer twice before analysis, incubated with activation cocktail (Biolegend) for 2 h, and mixed with monensin for 4 h. The stained cells were harvested, washed, re-suspended in fixation solution (500 μL/1 × 10^6^ cells), and incubated in dark at 4 °C for 20 mins. The fixed cells were washed with Intracellular Staining Perm Wash Buffer five times, stained with anti-IFN-γ, anti-IL-2 and anti-IL-4 antibodies at 37 °C for 30 mins, washed twice, and re-suspended in staining buffer for analysis. The LSRII flow cytometer (BD Biosciences) was used to analyze the expression of surface and intracellular markers for the T cell samples, and data were analyzed using FlowJo software (Tree Star). Gating was set where negative sample has < 0.5% fluorescent population.

### Statistical analysis

We used the T cells isolated from five donors over different stages of this study and collected data from triplicate bioreactor runs. Comparisons across donors were performed by analyzing the cell function and expansion capability. All data are presented as mean ± standard error of the mean (SEM). Two-tailed t tests were used to determine the significance difference between two groups. Multiple groups were analyzed by one-way ANOVA. Statistical significance with *p* value of ≤0.05 was considered for all tests.

## Abbreviations

CAR: Chimeric antigen receptor
DO: Dissolved oxygen
IL-2: Cytokine interleukin-2
KLRG1: Killer cell lectin-like receptor subfamily G member 1
PBMC: Peripheral blood mononuclear cell
TCR: T cell receptor
Th1: T-helper type 1
Th2: T-helper type 2
VCD: Viable cell density
